# PD-L1 expression as a potential predictor of immune checkpoint inhibitor efficacy and survival in patients with recurrent or metastatic nasopharyngeal cancer: a systematic review and meta-analysis of prospective trials

**DOI:** 10.3389/fonc.2024.1386381

**Published:** 2024-06-03

**Authors:** Ruyu Xu, Charlene H.L. Wong, Kenneth S.K. Chan, Chi Leung Chiang

**Affiliations:** ^1^ Department of Clinical Oncology, Li Ka Shing (LKS) Faculty of Medicine, The University of Hong Kong, Queen Mary Hospital, Hong Kong, Hong Kong SAR, China; ^2^ School of Nursing, Li Ka Shing (LKS) Faculty of Medicine, The University of Hong Kong, Queen Mary Hospital, Hong Kong, Hong Kong SAR, China

**Keywords:** nasopharyngeal carcinoma, recurrence or metastasis, PD-L1, immune checkpoint inhibitors, meta-analysis

## Abstract

**Background:**

The predictive value of programmed death-ligand 1 (PD-L1) expression in nasopharyngeal cancer (NPC) patients receiving immune checkpoint inhibitors (ICIs) remains controversial. This study aimed to evaluate the optimal threshold of PD-L1 expression in predicting the efficacy of ICIs in patients with recurrent or metastatic (R/M) NPC.

**Methods:**

A meta-analysis was performed by retrieving relevant literature from PubMed, EMBASE, and Cochrane Library databases. Data on the pooled risk ratio (RR), mean overall survival (OS), progression-free survival (PFS), overall response rate (ORR) with 95% confidence interval, and 1%, 10%, and 25% PD-L1 expression cutoff points were obtained to examine the role of PD-L1 as a biomarker in R/M NPC patients receiving immunotherapy.

**Results:**

In total, 1,312 patients from 14 studies were included. An improvement in PFS was observed in both patients with PD-L1 ≥ 1% (RR = 0.76, 95% CI 0.62–0.92, P = 0.005) and those with PD-L1 < 1% (RR = 0.68, 95% CI: 0.35–1.32, P = 0.26) who received first-line treatment with immunotherapy, with no significant difference between these subgroups. The pooled ORR was significantly higher in patients with PD-L1 ≥ 1% (ORR = 0.37) than in those with PD-L1 < 1% (ORR = 0.22) (P < 0.01) undergoing subsequent-line treatment. However, when we used the PD-L1 cutoff values of 10% and 25%, there was no significant difference between the positive (PD-L1 expression ≥ the cutoff value) and negative (PD-L1 expression < the cutoff value) subgroups. PD-L1 ≥ 1% also tended to be associated with better PFS and OS.

**Conclusions:**

Our meta-analysis suggested that first-line immunotherapy could significantly improve PFS in R/M NPC patients, regardless of the PD-L1 expression levels. Positive PD-L1 expression (≥ 1%) might be a potential predictive biomarker for a better overall response to immunotherapy in R/M NPC patients in subsequent-line setting.

**Systematic review registration:**

https://www.crd.york.ac.uk/prospero/display_record.php?ID=CRD42024495841 PROSPERO, identifier CRD42024495841.

## 1 Introduction

Nasopharyngeal carcinoma (NPC) is a common type of head and neck cancer with a skewed geographical, ethnic, and sex distribution. It is particularly prevalent in east and southeast Asia, where the highest age-standardized rates occur (1). According to GLOBOCAN 2020 data, approximately 133,354 new cases and 80,008 deaths from NPC were reported worldwide, of which 62,444 cases (46.8%) and 34,810 deaths (43.5%) were registered in China (2).

In the past decade, the global incidence and mortality rates of NPC have gradually declined (3), which could be attributable to lifestyle and environmental changes, the use of intensity-modulated radiation therapy, and the increasing application of adjuvant chemotherapy (4, 5). However, approximately 15%–30% of patients who develop recurrent or metastatic (R/M) NPC have a median overall survival (OS) of less than 2 years (6). The main challenges in treating these patients are overcoming chemo-resistance and reducing the risk of adverse events (7). Currently, immunotherapies, especially immune checkpoint inhibitors (ICIs), represent a promising strategy to resolve these problems and effectively treat R/M NPC patients.

ICIs, particularly anti-programmed death-1 (PD-1)/programmed death-ligand 1 (PD-L1) and anti-cytotoxic T-lymphocyte-associated antigen 4 (CTLA-4) antibodies, which activate CD8-positive T cells and induce cancer cell mortality, have revolutionized the treatment of advanced cancers. The tumor microenvironment of NPCs, characterized by massive inflammatory and immune cell infiltration, allows NPC patients to fully benefit from ICI therapy. ICIs have emerged as effective treatment options for patients with refractory R/M NPC. More recently, the Food and Drug Administration (FDA) approved toripalimab as a treatment for R/M NPC to be used in combination with first-line chemotherapies or subsequent-line monotherapies (8). The National Comprehensive Cancer Network (NCCN) Guidelines version 2.2024 refer to cisplatin/gemcitabine combined with ICIs as the first-line treatment in the management of R/M NPC (9). However, only about 50% of patients respond to treatment, indicating the major challenge of identifying patients who are suitable for immunotherapy (6).

The level of PD-L1 expression is one of the most commonly explored predictive biomarkers for the success of ICIs. Previous studies have shown that higher PD-L1 expression levels are associated with a higher response rate and better survival in patients with advanced stage melanoma treated with ICIs (10–13). However, the predictive value of PD-L1 expression in NPC patients receiving ICIs remains controversial (14–16). Currently, there is no report of studies exploring the optimal cutoff value of PD-L1 expression to guide the clinical use of ICIs.

In this systematic review (SR), we comprehensively evaluated whether the expression level of PD-L1 influences the efficacy of anti-PD-1/PD-L1 monotherapy or combined therapy in NPC patients. Furthermore, subgroup analyzes were performed to assess and quantify the best cutoff value for PD-L1-positive tumors to guide future clinical practice.

## 2 Materials and methods

The study was reported in accordance with the Preferred Reporting Items for Systematic Review and Meta-Analyzes (PRISMA) (17). The protocol for this SR and meta-analysis was registered in PROSPERO (no.: 495841).

### 2.1 Eligibility criteria

To be eligible for this SR, studies were required to satisfy the following Population, Intervention, Comparison, Outcomes, and Study design (PICOS) criteria. Patients with a pathological diagnosis of R/M NPC who received immunotherapy with/without other systematic treatments were included. The included studies were required to report at least one clinical outcome, namely OS, progression-free survival (PFS), or overall response rate (ORR), based on the PD-L1 expression levels of patients. Randomized control trials (RCTs) and non-RCTs were considered eligible. There was no restriction on the language or publication status of studies. Patients receiving radiotherapy were not eligible for this SR. Review articles, case reports, conference abstracts, protocols, editorials, and commentaries were also excluded.

### 2.2 Literature search

A comprehensive literature search was performed on PubMed, Embase, and the Cochrane Library to identify potential eligible studies published from January 2013 to December 6, 2023. We also manually searched for eligible studies by checking the reference lists of retrieved studies to minimize the risk of missing relevant information. The detailed search strategy is described in **Supplementary File 1**.

### 2.3 Literature selection

The titles and abstracts of potential studies were screened independently by two authors (C.H.L.W. and S.K.CH.), and then their full texts were assessed for eligibility. If there was any dispute, it was resolved through discussion between the two authors. A third author (C.L.C.) was consulted to settle unresolved disagreements.

A list of studies for inclusion was generated. For duplicate studies, the most recent and comprehensive version of each was selected for inclusion. SRs identified during the search were examined to ensure that no eligible studies were omitted.

### 2.4 Data extraction

Data were extracted by one author (R.Y.X.) and cross-reviewed by the other two authors (C.H.L.W. and S.K.CH.). Key information, including authors’ details, year of publication, study population, sample size, patient characteristics, follow-up time, intervention, and results of all prespecified outcomes, were extracted from each eligible study using a pre-designed data-extraction table.

### 2.5 Methodological quality assessment

The methodological quality of all included studies was evaluated by two reviewers (C.H.L.W. and R.Y.X.) independently using the Cochrane’s Risk of Bias in Randomized Trials (RoB 2) tool for RCTs (18) and the Cochrane’s Risk of Bias in Non-Randomized Studies of Interventions (ROBINS-I) tool for non-randomized studies (19). For the included single-arm non-randomized studies, risk of bias was assessed using a modified ROBINS-I approach (20). The risk of bias was categorized as low, moderate, serious, or critical. Publication bias was assessed using Egger’s regression test and through a visual inspection of funnel plot asymmetry if there were more than 10 studies (21).

### 2.6 Data analysis

To examine the role of PD-L1 among R/M NPC patients receiving immunotherapy with/without other systematic treatment, we conducted a pairwise random-effects meta-analysis comparing immunotherapy plus chemotherapy patients with controls in the first-line therapy setting using RevMan version 5.4. We used pooled risk ratios (RRs) with 95% confidence intervals (CIs) to present PFS data.

Single-arm random-effects meta-analyzes were performed to synthesize the effects of immunotherapy with/without other systematic treatments on the clinical outcomes (i.e. OS, PFS, and ORR) in both first-line and subsequent-line settings using R version 4.2.3. The pooled estimated mean OS and PFS, as well as the pooled ORR with 95% CI, are presented.

For both pairwise and single-arm meta-analyzes, subgroup analysis was performed on each clinical outcome by stratifying patients into two groups (1): PD-L1 positive and (2) PD-L1 negative. Three cutoff points for PD-L1 expression level were used: 1%, 10%, and 25%. We also conducted a sensitivity analysis of the impact of treatments on the clinical outcomes by excluding patients who received combined immunotherapies and targeted therapies. We used I^2^ values to quantify the level of heterogeneity, with I^2^ < 25% indicating a low level of heterogeneity, 25%–50% indicating a moderate level of heterogeneity, and >50% indicating a high level of heterogeneity (22).

## 3 Results

### 3.1 Literature search and selection

**Figure 1:**
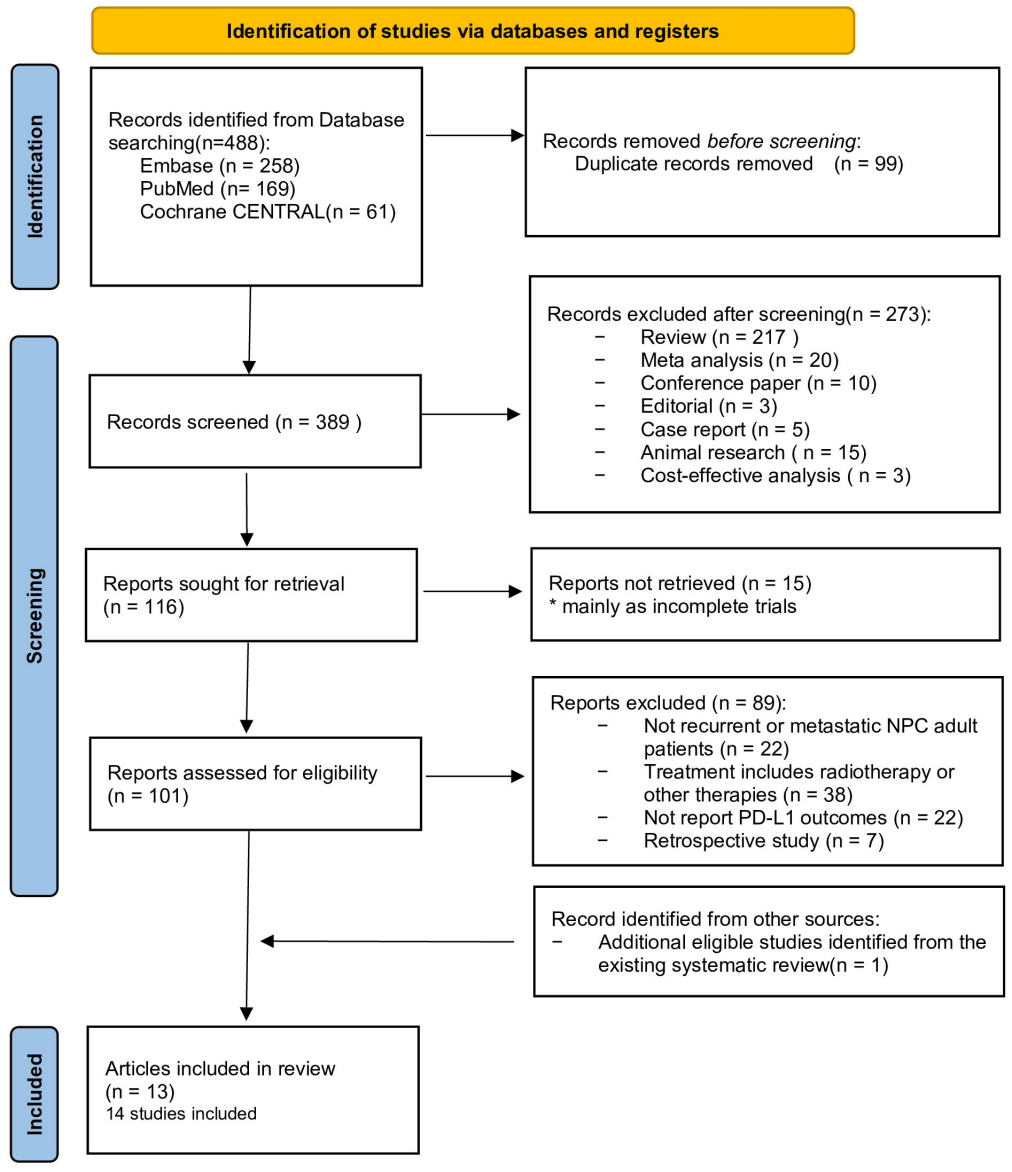
Flow diagram of meta-analysis for inclusion/exclusion of studies.

The literature search yielded 488 citations, among which 99 duplicate studies were removed. After screening the titles and abstracts, 116 eligible articles remained. As the full texts of 15 articles were not available, only 101 remaining papers proceeded to full-text assessment. Eighty-nine of these were excluded because (i) no recurrent or metastatic NPC adult patients were included (n = 22); (ii) treatment included radiotherapy or other therapies (n = 38); (iii) PD-L1 outcomes were not reported (n = 22); or (iv) they were retrospective studies (n = 7). With the identification of one additional reference through manual searches of the reference lists of included studies, a total of 14 studies in 13 articles were included in this systematic review. Details of the literature search and study selection are shown in the PRISMA flow diagram (**Figure 1**) (23).

### 3.2 Characteristics of the included studies

Basic information on the qualified studies analyzed in this meta-analysis is available in **Table 1**. One study was an RCT (24), two were non-randomized studies (25, 29), and 11 were single-arm studies reported in 10 articles (26–28, 30–36). All of the included studies were published between 2017 and 2023, with a majority of them conducted on Asian patients (24–28, 30–36). The total sample size of the included studies was 1,434 patients, with six studies having a sample size of more than 100 (24, 25, 28, 29, 31, 32). The follow-up period ranged from 1.0 to 2.5 years. Five studies used combined therapy as the intervention (24, 25, 33, 34, 36), while eight studies treated patients with mono-immunotherapy (26–32, 35). These studies used different PD-L1 measurements, with four of them using 22C3 (26, 29, 30, 36) (full details are provided in **Table 2**). Among all of these studies, 11 reported ORR (26–36), while 10 reported PFS (24, 25, 27, 29–34, 36) and five reported OS (27, 29–32) based on PD-L1 expression. The overall risk of bias of eight studies (61.5%) (24–27, 30, 32, 35, 36) was considered low, but that of five studies (38.5%) was moderate (28, 29, 31, 33, 34), four of which were due to missing data (**Supplementary Table 1**).

**Table 1:** Characteristics of the studies included in the meta-analysis.

Studies	Line of treat-ment	Type of study	Region & population	Sample (Number of patients tested PD-L1)	Male (%)	Median age (range)	Follow-up (years)	Treatment	Top three most common adverse events	Outcomes
24 (JUPITER-02)	1st	Prospective (phase III)	China, Asian (100%)	146 (130)	124 (85)	46 (19–72)	2	Toripalimab 240 mg (day 1), gemcitabine 1 g/m2 (Days 1 and 8), and cisplatin 80 mg/m2 (day 1) every 3 weeks	Leukopenia 91.1%, Anemia 88.4%, Neutropenia 85.6%	PFS
				143 (133)	116 (81)	51 (21–72)		Placebo(day 1), gemcitabine 1 g/m2 (Days 1 and 8), and cisplatin 80 mg/m2 (day 1) every 3 weeks	Leukopenia 94.4%, Anemia 94.4%, Neutropenia 93.0%	
25 (RATIONALE 309)	1st	Prospective (phase III)	China, Asian (100%)	131 (123)	103 (78.6)	50 (26–74)	2	Tislelizumab 200 mg (day 1), gemcitabine 1 g/m2(Days 1 and 8), and Cisplatin 80 mg/m2 (day 1) every 3 weeks	Anemia 87.8%, WBC decreased 61.8%, Neutropenia 60.3%	PFS
				132 (119)	103 (78.0)	50 (23–73)		Placebo (day 1), gemcitabine 1 g/m2(Days 1 and 8), and cisplatin 80 mg/m2 (day 1) every 3 weeks	Anemia 89.4%, Nausea 70.5%, WBC decreased 61.4%	
26 (NCI-9742)	2nd or later	Prospective (phase II)	Hong Kong, Asian (82.2%)	45 (42)	35 (77.8)	57 (37-76)	2	Nivolumab 3 mg/kg every 2 weeks	Fatigue 33%, Hypothyroidism 13%, AST level increased 13%	ORR
27 (KEYNOTE-028)	2nd or later	Prospective (phase Ib)	Hong Kong, Asian (63.0%)	27	21 (77.8)	52 (18–68)	2	Pembrolizumab 10 mg/kg every 2 weeks	Rash 25.9%, Pruritus 25.9%, Pain 22.2%	OS, PFS, ORR
28 (POLARIS-02)	2nd or later	Prospective (phase II)	China, Asian (100%)	190 (182)	158 (83.2)	46.4 (22–71)	2.5	Toripalimab 3mg/kg every 2 weeks	Hypothyroidism 23.7%, Anemia 15.3%, AST increased 15.3%	ORR
29 (KEYNOTE-122)	2nd or later	Prospective (phase III)	world	117	98 (83.8)	51 (42-59)	2	Pembrolizumab 200 mg every 3 weeks	Hypothyroidism 13.8%, Fatigue 12.1%, Rash 11.2%	OS, PFS, ORR
				116	95 (81.9)	53 (46.5–61)		Capecitabine 1000 mg/m2, gemcitabine 1250 mg/m2 or docetaxel 75 mg/m2 every 3 weeks	Neutropenia 34.8%, Anemia 25.9%, Palmar-plantar erythrodysesthemia syndrome 19.6%	
30 (M7824)	2nd or later	Prospective (phase II)	Hong Kong, Asian (100.0%)	38 (31)	33 (86.8)	54 (18–72)	1.5	Bintrafuspalfa 1200 mg every 2 weeks	Anemia 50%, Pruritus 36.8%, Rash 31.6%	OS, PFS, ORR
31 (KL-A167)	2nd or later	Prospective (phase II)	China, Asian (100%)	132 (127)	109 (82.6)	49 (26−68)	2	KL-A167 900mg every 2 weeks	Hypothyroidism 13.1%, WBC decrease 10.5%, AST increase 9.2%	OS, PFS, ORR
32 (CAPTAIN)	2nd or later	Prospective (phase II)	China, Asian (100%)	156 (150)	124 (79.5)	48 (23–71)	2	Camrelizumab 200mg every 2 weeks	RCEP 89.7%, Anemia 27.6%, Hypothyroidism 24.4%	OS, PFS, ORR
33	2nd or later	Prospective (phase II)	China, Asian (100%)	40 (29)	32 (80.0)	49 (37–54)	2	Camrelizumab 200 mg every 3 weeks plus oral apatinib 250 mg daily	Hypothyroidism 68.1%, Hypertension 66.7%, Leukopenia 61.1%	PFS, ORR
33				32 (23)	24 (75.0)	40(36–50)		Apatinib in the first 2 weeks, then camrelizumab plus apatinib.		
34	2nd or later	Prospective (phase II)	China, Asian (100%)	58 (47)	46 (79.3)	NA	2	Apatinib 250 mg daily and camrelizumab 200 mg every 3 weeks	Hypertension 70.7%, Dysphagia 69.0%, Pharyngolaryngeal pain 67.2%	PFS, ORR
35	2nd or later	Prospective (phase I/II)	China, Asian (100%)	21 (20)	NA	NA	2	Tislelizumab 200mg every 3 weeks	Anemia 7.7%, AST increase 7.7%, ALT increase 6.3%	ORR
36	2nd or later	Prospective (phase II)	China, Asian (100%)	18	15 (83.3)	47	2	Famitinib 20 mg daily and camrelizumab 200 mg every 3 weeks	Neutropenia 66.7%, Albuminuria 61.1%, Leukopenia 61.1%	PFS, ORR

PD-L1, programmed cell death ligand-1; NA, not reported; IV, intravenous injection; WBC, white blood cell; AST, aspartate aminotransferase; RCEP, reactive capillary endothelial proliferation; ALT, alanine aminotransferase.

**Table 2:** Technical information of PD-L1 measurement in the included studies.

Potential studies	Line of treatment	Sample type	PD-L1 measurement	Antibody(Company/Source/Clone)	PD-L1 status (Definition of positivity, TC/IC)
Mai et al., 2021 (JUPITER-02)	1st	Fresh or archival tumor tissue samples	Tumor cell membrane IHC staining	Ventana Benchmark Ultra, rabbit, JS311	PD-L1 +(≥1% TC/IC)
25 (RATIONALE 309)	1st	Fresh or archival tumor tissue samples	Tumor cell membrane IHC staining	Ventana Medical Systems, SP263	PD-L1+(>1% TC) PD-L1h(>10% TC)
26 (NCI-9742)	2nd or later	Fresh or archival tumor tissue samples	Tumor cell membrane IHC staining	Agilent Technologies, 22C3	PD-L1 +(≥1% TC/IC)
27 (KEYNOTE-028)	2nd or later	Fresh or archival tumor tissue samples	Tumor cell membrane IHC staining	QualTek Molecular Laboratories	PD-L1 +(≥1% TC/IC)
28 (POLARIS-02)	2nd or later	NA	Tumor cell membrane IHC staining	SP142	PD-L1+(>1%/25% TC)
29 (KEYNOTE-122)	2nd or later	NA	Tumor cell membrane IHC staining	Agilent Technologies, 22C3	PD-L1+(>1%/10%/20% CPS)
30 (M7824)	2nd or later	NA	Tumor cell membrane IHC staining	22C3	PD-L1 +(≥1%/25% TC)
Shi et al., 2023 (KL-A167)	2nd or later	NA	enzyme-linked immu nosorbent assay (ELISA)	SAB-028	PD-L1+(>1% TC)
32 (CAPTAIN)	2nd or later	Fresh or archival tumor tissue samples	Tumor cell membrane IHC staining	Abcam SP142	PD-L1+(>1%/10%/20% TC)
33	2nd or later	Fresh or archival tumor tissue samples	Tumor cell membrane IHC staining	Abcam, ab205921	PD-L1+(>1% **TPS**/CPS)
33					
34	2nd or later	NA	Tumor cell membrane IHC staining	CST13684	PD-L1+(>1%/10%/25% CPS)
35	2nd or later	NA	NA	SP263	PD-L1+(>10% TC)
36	2nd or later	Fresh or archival tumor tissue samples	Tumor cell membrane IHC staining	Agilent Technologies, 22C3	PD-L1+(>1% CPS)

TC, tumor cells; IC, tumor-infiltrating immune cells; TPS, tumor cell proportion score; CPS, combined positive score; IHC, immunohistochemistry.

### 3.3 Results of first-line treatment

**Figure 2:**
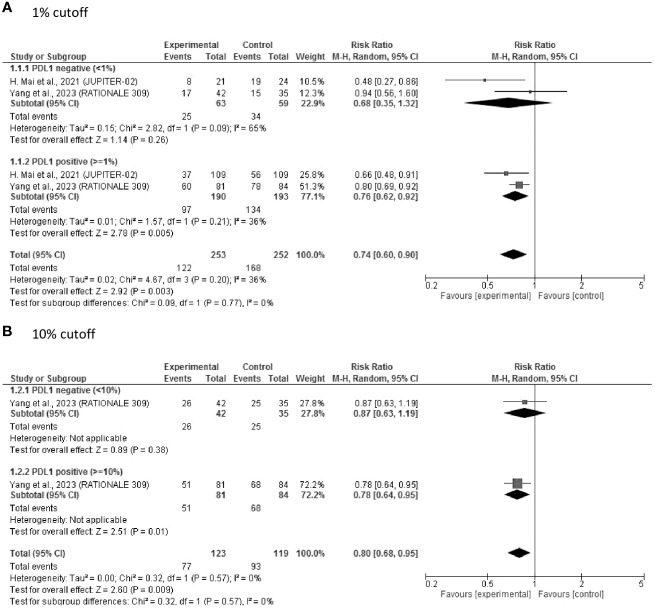
Forest plot of RR of PFS after first-line treatment. **(A)** 1% cut off; **(B)** 10% cutoff. PDL1, programmed cell death ligand-1; RR, risk ratio; PFS, progression-free survival.

Two studies of first-line therapy that included 505 patients reported PD-L1 levels and related PFS outcomes. As depicted in **Figure 2A**, the pooled results showed that ICIs significantly prolonged PFS (RR = 0.74, 95% CI: 0.60–0.90, P = 0.003). An improvement in PFS was observed in both patients with PD-L1 ≥ 1% (RR = 0.76, 95% CI 0.62–0.92, P = 0.005) and those with PD-L1 < 1% (RR = 0.68, 95% CI: 0.35–1.32, P = 0.26), with no significant difference between these subgroups. When using the PD-L1 cutoff of 10%, which was only used in the “RATIONAL 309” study, there was a tendency toward better PFS in PD-L1-positive patients, with RRs of 0.78 (95% CI: 0.64–0.95, P = 0.01) and 0.87 (95% CI: 0.63–1.19, P = 0.38) for PD-L1 ≥ 10% and PD-L1 < 10%, respectively (**Figure 2B**).

### 3.4 Results of subsequent-line treatments

Twelve studies with 929 patients were included in this meta-analysis of first- or subsequent-line treatment. The PD-L1 levels reported in these studies were graded using different standards (PD-L1-positive at > 1% TC/IC (n = 10) (26–28, 30–36), PD-L1-positive at > 10% TC/IC (n = 4) (29, 32, 34, 35), and PD-L1 positive at > 25% TC/IC (n = 3) (28, 30, 34).

#### 3.4.1 ORR of PD-L1 status after subsequent-line treatment

**Figure 3:**
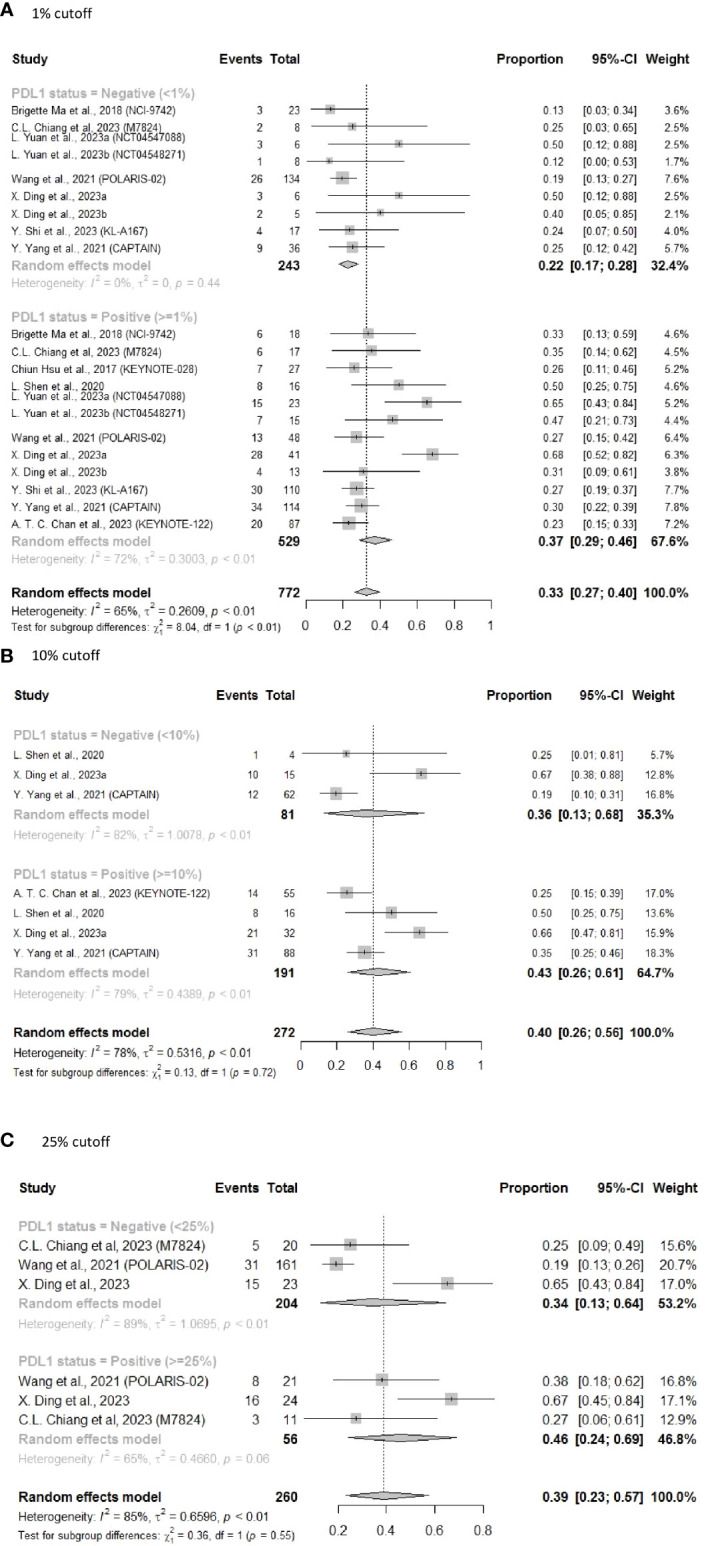
Forest plot of pooled results of ORR following subsequent-line treatment. **(A)** 1% cutoff; **(B)** 10% cutoff; **(C)** 25% cutoff.

The forest plots show that the pooled ORR was significantly higher for NPC patients with PD-L1 ≥ 1% (ORR = 0.37, 95% CI: 0.29–0.46) than for those with PD-L1 < 1% (ORR = 0.22, 95% CI: 0.17–0.28) (subgroup difference, P < 0.01) (**Figure 3A**).

Using a PD-L1 cutoff value of 10% resulted in a similar pattern (**Figure 3B**) (ORR = 0.43, 95% CI: 0.26–0.61 vs ORR = 0.36, 95% CI: 0.13–0.68). Using the 25% threshold for PD-L1 also revealed similar findings, with an ORR of 0.46 (95% CI: 0.24–0.69) in the PD-L1 ≥ 25% subgroup vs an ORR of 0.34 (95% CI: 0.13–0.64) in the PD-L1 < 25% subgroup (**Figure 3C**). However, as a result of the limited sample sizes and significant heterogeneity, differences between the subgroups were not statistically significant. We also noticed that the ORR appeared to rise with increasing PD-L1 expression level, suggesting that the efficacy of ICIs in NPC patients was correlated with PD-L1 expression levels.

**Figure 4:**
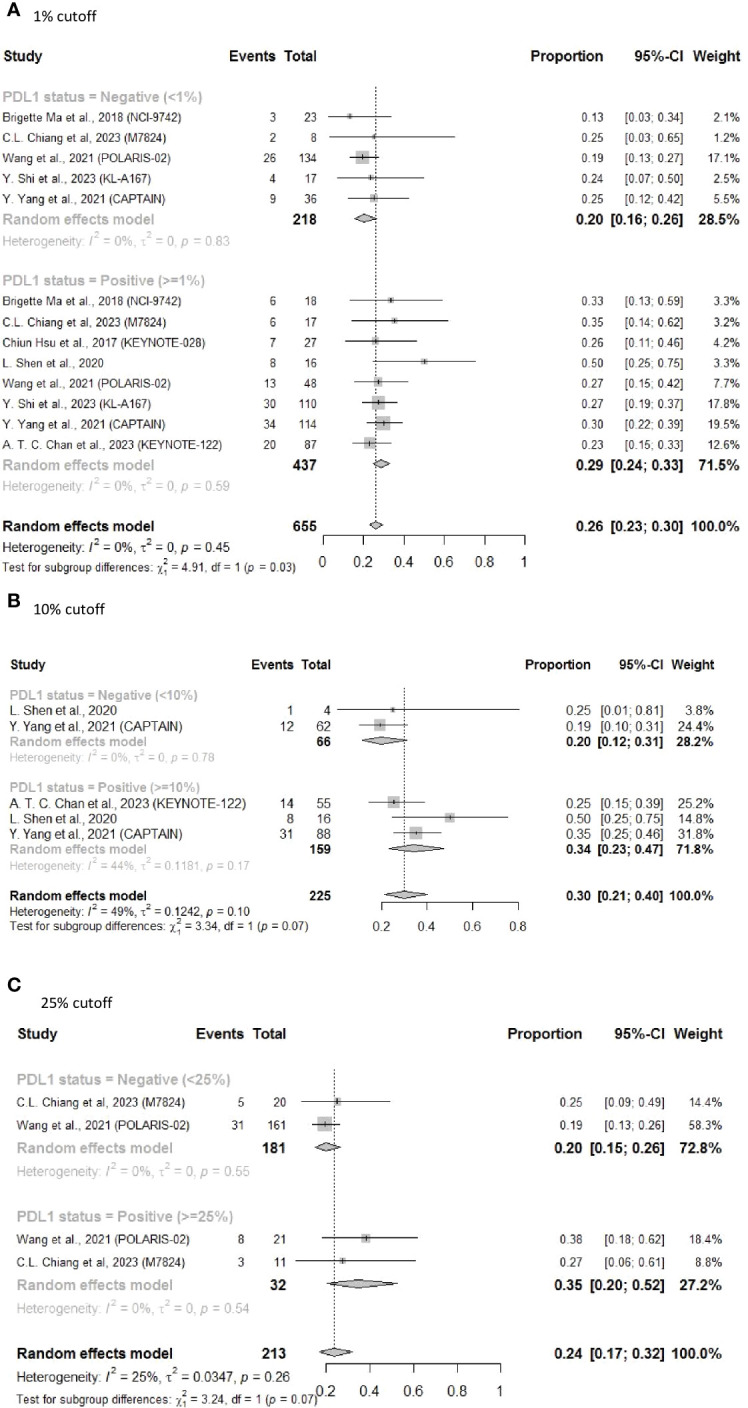
Forest plot of ORR in sensitivity analysis excluding patients who received combined immunotherapy and targeted therapy as subsequent-line treatments. **(A)** 1% cutoff; **(B)** 10% cutoff; **(C)** 25% cutoff.

To further elucidate the heterogeneity among these studies, sensitivity analysis was performed by excluding patients who received combined immunotherapy and targeted therapy. The results still showed a better ORR (0.29 vs 0.20) for PD-L1-positive patients who received ICI monotherapy, with significantly reduced heterogeneity (I^2 ^= 0% in both the groups, subgroup difference P = 0.03) (**Figure 4A**). Additionally, ORR improvement was more pronounced in the PD-L1-positive group vs the PD-L1-negative group for subsequent-line ORR with PD-L1 status of 10% (ORR = 0.34, 95% CI: 0.23–0.47 vs ORR = 0.20, 95% CI: 0.12–0.31, P = 0.07) (**Figure 4B**) and for subsequent-line ORR with PD-L1 status of 25% (ORR = 0.35, 95% CI: 0.20–0.52 vs ORR = 0.20, 95% CI: 0.15–0.26, P = 0.07) (**Figure 4C**).

#### 3.4.2 PFS and OS association with PD-L1 status after subsequent-line treatment

**Figure 5:**
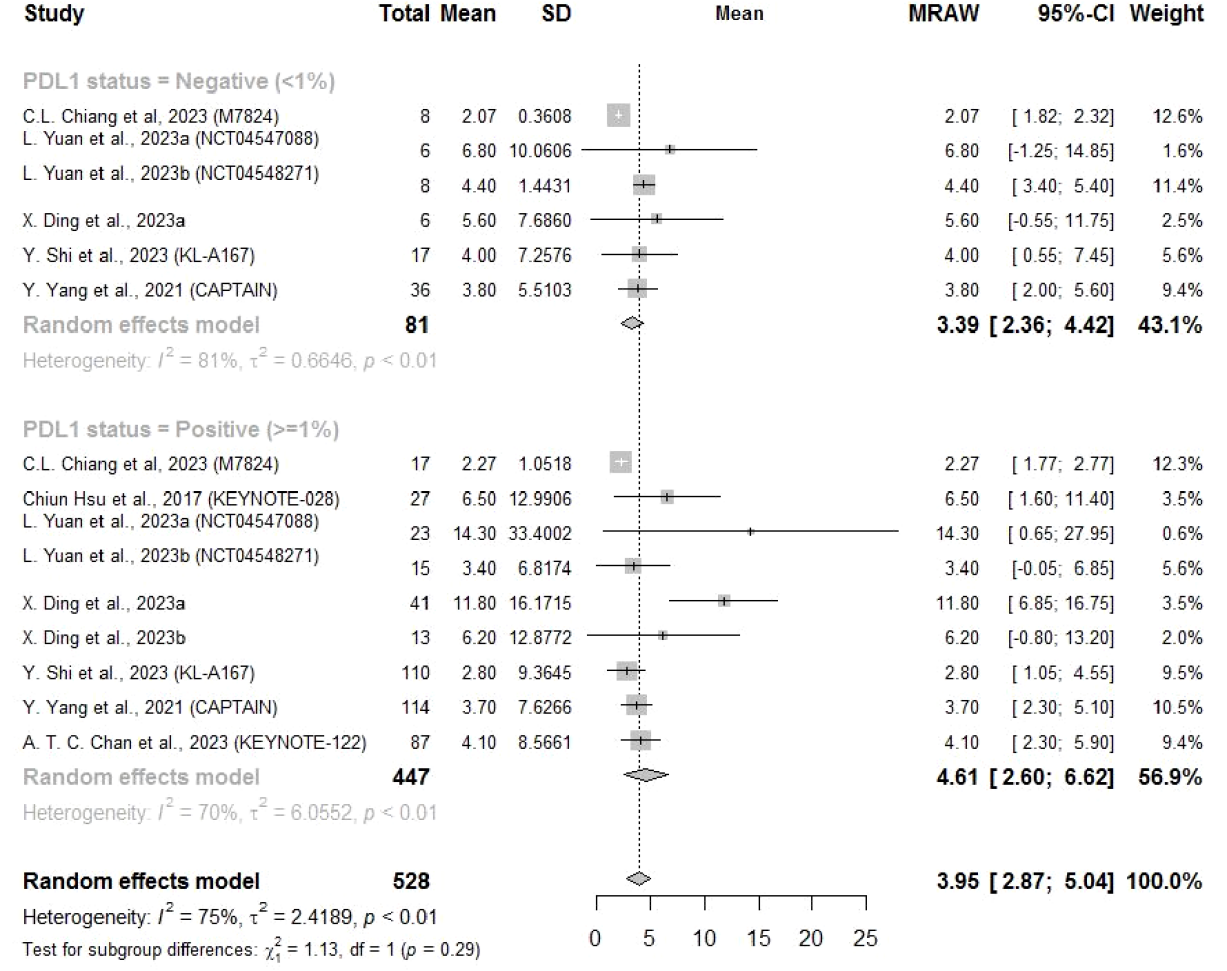
Forest plot showing pooled results of PFS after subsequent-line treatment (1% cutoff).

**Figure 6:**
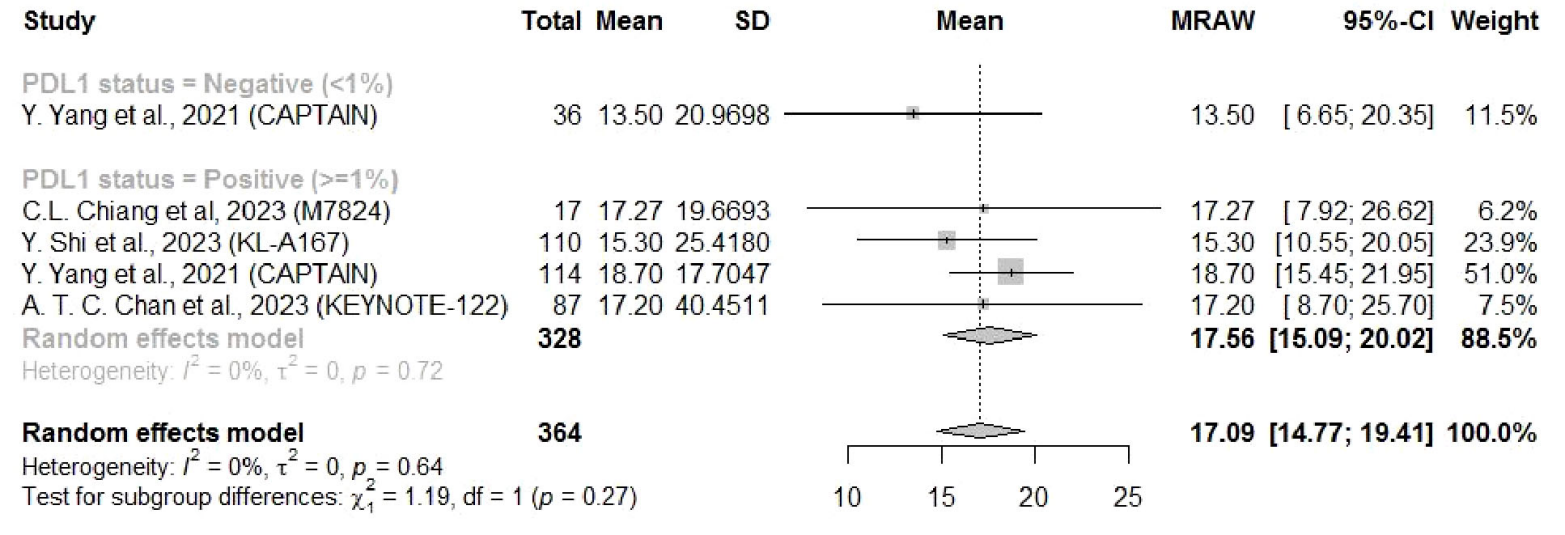
Forest plot of pooled results of OS after subsequent-line treatment (1% cutoff).

Eight studies reported the PFS and four reported the OS related to a PD-L1 cutoff of 1%. The PFS and OS results showed similar findings that both mean PFS (4.61 months, 95% CI: 2.60–6.62) and OS (17.56 months, 95% CI: 15.09–20.02) for NPC patients with PD-L1 ≥ 1% were longer than those for patients with PD-L1 < 1% (PFS: 3.39 months, 95%CI: 2.36–4.42; OS: 13.5 months, 95% CI: 6.65–20.35), but there was no significant subgroup difference (**Figures 5**, **6**).

### 3.5 Publication bias

**Figure 7:**
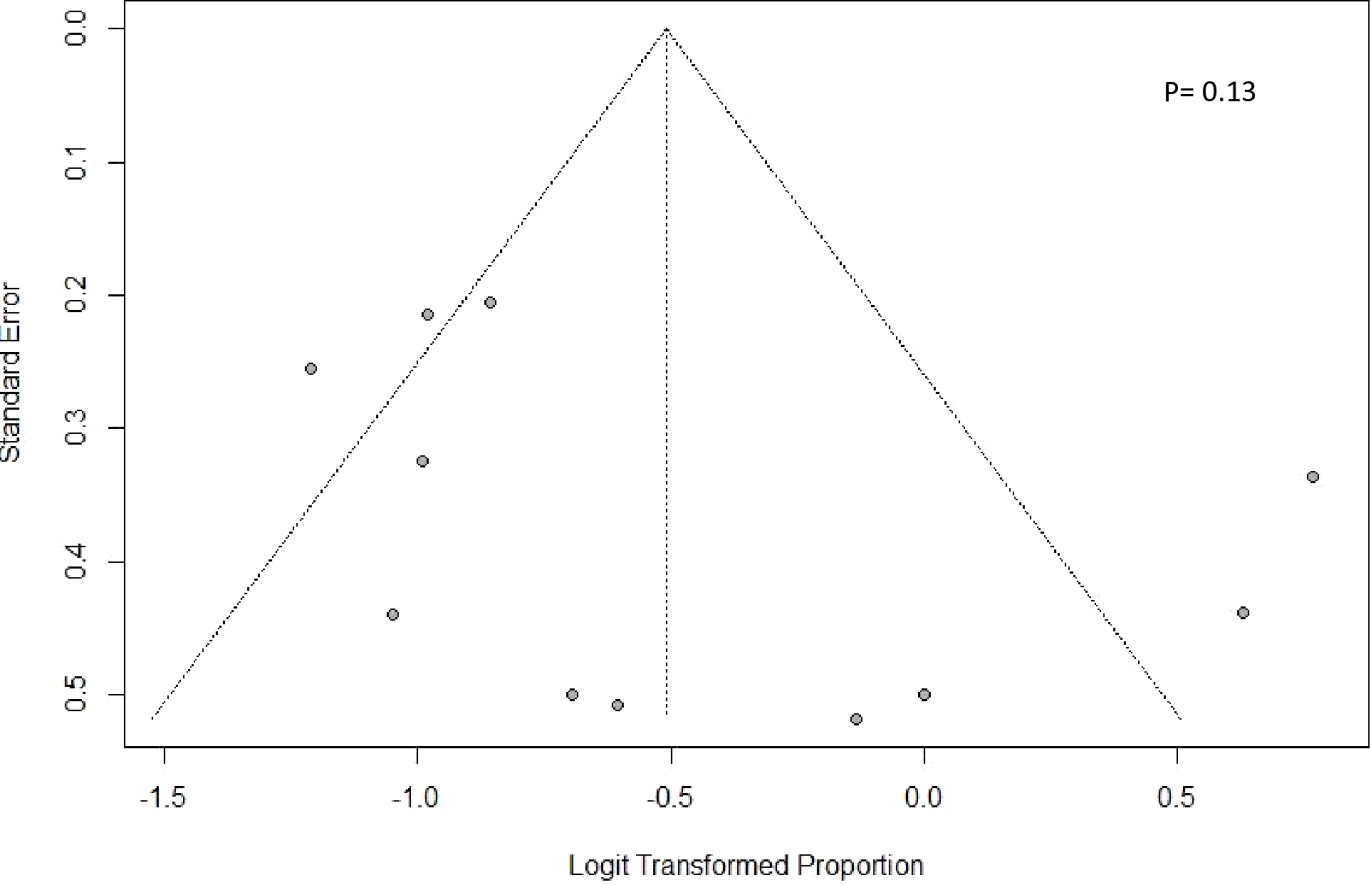
Funnel plot of publication bias.

Publication bias was assessed with an Egger’s regression plot for 12 articles focusing on subsequent-line therapy. The plot revealed no presence of publication bias (P = 0.13), and no asymmetry was found in the funnel plot (**Figure 7**).

## 4 Discussion

To the best of our understanding, this is the first in-depth analysis of the predictive value of PD-1/PD-L1 status in clinical trials of immunotherapy and combined therapy for patients with advanced metastatic NPC. We comprehensively evaluated the correlation between different expression levels of PD-L1 and the ORR, PFS, and OS of R/M NPC patients, with data retrieved from 14 studies that included 1,434 patients in total. We aimed to determine the predictive value of PD-L1 and to identify an optimal PD-L1 cutoff value for the selection of patients likely to respond effectively to anti-PD-1/PD-L1 treatment.

The NCCN guidelines only recommend to use i) cisplatin/gemcitabine alone, or ii) cisplatin/gemcitabine plus toripalimab, or iii) cisplatin/gemcitabine plus the other PD-1 inhibitors (pembrolizumab or nivolumab) as first-line therapies for R/M NPC (9). In our analysis of the two included studies in the first-line setting, the use of ICIs could significantly improve PFS, regardless of the PD-L1 expression levels. Various ICI monotherapies (toripalimab, pembrolizumab or nivolumab) are recommended as subsequent-line therapy based on the PD-L1 expression levels in NCCN guidelines (9). The availability of ample subsequent-line treatment data gave us the opportunity to draw more precise and accurate conclusions. The most compelling finding in our study was that patients with PD‐L1 ≥ 1% who received ICI in the subsequent-line setting had significantly higher ORR than in those with PD-L1 < 1%. Our pooled results showed no significant difference between subgroups in analysis of PFS and ORR for PD-L1 cutoff value of ≥ 10%, and ≥ 25%. However, higher the PD-L1 expression, the higher the probability that the patient was able to achieve clinical benefit from ICIs in the subsequent-line setting.

ICIs, which reactivate immune response in the tumor by preventing immunosuppressive factors from binding to their ligands, have fewer side effects compared to chemotherapy (37). Side effects of ICIs are usually mild. The most common side effects include fatigue, itchy rash, and diarrhea (38). In addition, as ICIs may also activate autoreactive T cells, they increase the risk of immune-related adverse events (irAEs). In our included studies, irAEs such as hypothyroidism, aspartate aminotransferase(AST) level increased, and rash, were also frequently reported. The detail information is shown in **Table 1**.

PD-L1, the most common immunosuppressive ligand, expressed on the tumor cell membrane combines with the PD-1 of tumor-infiltrating lymphocytes (TIL), contributing to tumor cell evasion from host immune system surveillance (39). In previous studies, high expression of PD-L1 appeared to adversely affect the survival outcomes of NPC patients. A meta-analysis involving 13 studies showed that PD-L1 over-expression in NPC was associated with a poor OS (hazard ratio = 1.48, 95% CI: 1.00–2.18, P = 0.049) (40). Another study discovered a significant correlation between high PD-L1 expression and a short PFS/OS (41). In contrast to previous studies that examined the prognostic value of PD-L1 in patients with NPC, our study evaluated the predictive value of PD-L1 expression for ICI therapy. The results provide evidence that PD-L1-positive patients received more benefit than PD-L1-negative patients at a PD-L1 cutoff value of 1%, which sets a preliminary framework for the R/M NPC patient population suitable for ICI treatment. However, as the cutoff values varied across articles, coupled with the fact that 1% was the most widely used expression-level cutoff for PD-L1 detection, more comprehensive studies on PD-L1 expression levels and ICI treatment efficacy are warranted to accurately validate these results.

Moreover, the PD-L1 expression on ICIs effect shows differences between first-line treatment and subsequent-line treatment, which may be caused by many factors. First, it is known that tumor progression is influenced by the tumor immune microenvironment, one of the important mechanisms is escape from immune surveillance with the selection of poorly immunogenic cells (42, 43). When the disease becomes refractory, the tumor microenvironment (TME) becomes more immune-suppressive. As a result, in the first-line setting when TME is still favorable, the immunotherapy-chemotherapy combination would improve survival regardless of PD-L1 expression. However, in subsequent line settings when TME becomes more immunosuppressive, only those with higher PD-L1 expression derived benefit from checkpoint inhibitors.

Second, all first-line trials evaluate the immunotherapy-chemotherapy combination while most later-line studies are using immunotherapy-alone (44). Chemotherapy could activate the T-cell priming and recruitment and works synergistically with immunotherapy, therefore patients who accept first-line treatment of immunotherapy-chemotherapy combination would respond to the treatment regardless of PD-L1 expression.

Though PD-L1 is the most widely studied biomarker for immunotherapy, additional biomarkers have been evaluated in several studies. For instance, a meta-analysis showed that patients with lower baseline plasma Epstein-Barr virus (EBV) DNA levels had a higher ORR and longer median PFS than those with higher EBV DNA levels, but tumor mutational burden (TMB) was not significantly correlated with clinical prognosis in NPC patients treated with ICIs (16). Furthermore, a statistical difference in PFS was observed between patients with tumors showing loss of HLA-A and/or HLA-B expression, and patients with tumors expressing both HLA-A and HLA-B in trial NCI-9742 (26). A single-arm phase II clinical trial indicated that, in R/M NPC patients, a strong suppression of TGFβ1 levels was associated with worse ORR and PFS (30).

With the development of bioinformatics and biotechnologies, novel forms of biomarkers, such as mutations/chromosomal abnormalities, have been made available that provide new perspectives on precision medicine. A recent clinical trial revealed that 11q13.3 focal amplification and high MRGPRF expression are predictive of poor outcomes following gemcitabine plus apatinib and toripalimab therapy, but in another study (POLARIS-02), the genomic alternations had no statistically significant associations with clinical efficacy (28, 33). However, our study of the PD-L1 biomarker has particular clinical relevance. PD-L1 status is readily used in clinical settings, as the technology is well established and inexpensive.

Our meta-analysis has several limitations. First, there was significant variability in the literature with regards to the prevalence and prognostic significance of PD-L1 expression in NPC patients, probably because of differences in the assays and scoring methods used across studies. However, in a cross-correlation study performed using different PD-L1 immunohistochemical assays, the JS311 antibody had similar PD-L1 staining patterns and scores to the antibodies 22C3, 28–8, and SP263 (45). The predictive utility of PD-L1 expression may also depend on its differential expression in immune cells versus tumor cells. Second, there was a lack of sufficient clinical trials of first-line treatments reporting OS and ORR in patients with different PD-L1 expression levels that could be included in our analysis. Despite the encouraging outcomes, the limited number of articles means we are skeptical of the conclusions, and more clinical trials focusing on ICI treatments are needed for further validation. Third, only three and two studies were included in the analysis of the PFS and OS, respectively, for the PD-L1 10% level. More clinical trials are needed to further enrich and validate our conclusions and better guide the use of clinical PD-L1 levels to maximize the benefits and reduce the side effects of ICIs. Lastly, most of the studies included were conducted in Asian populations, and the regional characteristics of NPC may limit the generalizability of our findings.

## 5 Conclusions

Our meta-analysis suggested that first-line immunotherapy could significantly improve PFS in R/M NPC patients, regardless of the PD-L1 expression levels. Nonetheless, positive PD-L1 expression (≥ 1%) might be a potential predictive biomarker for a better response to immunotherapy in R/M NPC patients in subsequent-line setting. The higher the PD-L1 expression, the higher the probability that the patient was able to achieve clinical benefit from subsequent-line treatment.

## Data availability statement

The original contributions presented in the study are included in the article/**Supplementary Material**. Further inquiries can be directed to the corresponding author.

## Author contributions

RX: Data curation, Investigation, Methodology, Visualization, Writing – original draft, Writing – review & editing. CW: Data curation, Formal analysis, Investigation, Methodology, Software, Validation, Writing – review & editing. KC: Investigation, Methodology, Validation, Writing – review & editing. CC: Conceptualization, Funding acquisition, Project administration, Supervision, Writing – review & editing.
