# Supplementary material for: PD-L1 expression as a potential predictor of immune checkpoint inhibitor efficacy and survival in patients with recurrent or metastatic nasopharyngeal cancer: a systematic review and meta-analysis of prospective trials
Source: Front Oncol. 2024 Jun 3;14:1386381. doi: 10.3389/fonc.2024.1386381 (PMC11180873; doi:10.3389/fonc.2024.1386381)
Supplement: Supplementary file 1 [file DataSheet_1.zip › Supplementary File 1.docx]

Key words

1, "nasopharyngeal carcinoma".mp.

2, "nasopharyngeal cancer".mp.

3, "nasopharynx carcinoma".mp.

4, "nasopharynx cancer".mp.

5, "immunotherapy".mp.

6, "programmed death-1".mp.

7, "PD-L1".mp.

8, "PD-1".mp.

9, "programmed death-ligand 1".mp.

10, "Immune checkpoint inhibitors".mp.

11, 1 or 2 or 3 or 4

12, limit 11 to yr="2013 -Current"

13, 5 or 6 or 7 or 8 or 9 or 10

14, limit 13 to yr="2013 -Current"

15, "trial".mp.

16, 11 and 13 and 15

Embase

| 1 | "nasopharyngeal carcinoma".mp. | 20902 |
| --- | --- | --- |
| 2 | "nasopharyngeal cancer".mp. | 3587 |
| 3 | "nasopharynx carcinoma".mp. | 21573 |
| 4 | "nasopharynx cancer".mp. | 5852 |
| 5 | "immunotherapy".mp. | 305632 |
| 6 | "programmed death-1".mp. | 89905 |
| 7 | "PD-L1".mp. | 52992 |
| 8 | "PD-1".mp. | 57133 |
| 9 | "programmed death-ligand 1".mp. | 10459 |
| 10 | "Immune checkpoint inhibitors".mp. | 31278 |
| 11 | 1 or 2 or 3 or 4 | 31170 |
| 12 | limit 11 to yr="2013 -Current" | 17871 |
| 13 | 5 or 6 or 7 or 8 or 9 or 10 | 374711 |
| 14 | limit 13 to yr="2013 -Current" | 275410 |
| 15 | "trial".mp. | 2668878 |
| 16 | 11 and 13 and 15 | 405 |
| 17 | limit 16 to embase status | 258 |

Pubmed

((nasopharyngeal carcinoma) OR (nasopharyngeal cancer) OR (nasopharynx carcinoma) OR (nasopharynx cancer)) AND ((immunotherapy) OR (programmed death-1)OR(PD-L1)OR(PD-1 )OR (programmed death-ligand 1 )) AND(trial) 169

Cochrane CENTRAL

#1 (nasopharyngeal carcinoma):ti,ab,kw OR (nasopharyngeal cancer):ti,ab,kw OR (nasopharynx carcinoma):ti,ab,kw OR (nasopharynx cancer):ti,ab,kw (Word variations have been searched) 2381

#2 (immunotherapy):ti,ab,kw OR (programmed death-1):ti,ab,kw OR (Immune checkpoint inhibitors):ti,ab,kw OR (programmed death-ligand 1):ti,ab,kw (Word variations have been searched) 15548

#3 #1 AND #2 with Cochrane Library publication date Between Jan 2013 and Dec 2023 62
