# Supplementary material for: PD-L1 expression as a potential predictor of immune checkpoint inhibitor efficacy and survival in patients with recurrent or metastatic nasopharyngeal cancer: a systematic review and meta-analysis of prospective trials
Source: Front Oncol. 2024 Jun 3;14:1386381. doi: 10.3389/fonc.2024.1386381 (PMC11180873; doi:10.3389/fonc.2024.1386381)
Supplement: Supplementary file 1 [file DataSheet_1.zip › Supplementary Table 1.docx]

| items | study | Bias due to randomization pocess | Bias due to deviations from intended interventions | Bias due to missing data | Bias in measurement of outcomes | Bias in selection of the reported results | overall bias |  |  |
| --- | --- | --- | --- | --- | --- | --- | --- | --- | --- |
| 1 | JUPITER-02 | low | low | low | low | low | low |  |  |
| items | study | Bias due to confounding | Bias in selection of participants | Bias in classification of interventions | Bias due to deviations from intended interventions | Bias due to missing data | Bias in measurement of outcomes | Bias in selection of the reported results | overall bias |
| 2 | RATIONALE 309 | low | low | low | low | low | low | low | low |
| 3 | NCI-9742 | low | low | NI | low | low | low | low | low |
| 4 | KEYNOTE-028 | low | low | NI | low | low | low | low | low |
| 5 | POLARIS-02 | low | low | NI | low | low | low | moderate | moderate |
| 6 | KEYNOTE-122 | low | low | low | low | moderate | low | low | moderate |
| 7 | M7824 | low | low | NI | low | low | low | low | low |
| 8 | KL-A167 | low | low | NI | low | moderate | low | low | moderate |
| 9 | CAPTAIN | low | low | NI | low | low | low | low | low |
| 10 | L. Yuan et al., 2023 | low | low | NI | low | moderate | low | low | moderate |
| 11 | X. Ding et al., 2023a | low | low | NI | low | moderate | low | moderate | moderate |
| 12 | L. Shen et al., 2020 | low | low | NI | low | low | low | low | low |
| 13 | X. Ding et al., 2023b | low | low | NI | low | low | low | low | low |

Supplementary Table 1. Quality assessment of the studies included in the meta-analysis.
